# Atypical Case of Takayasu Aortitis Presenting as Embolic Stroke With Concomitant Massive Thoracic Artery Aneurysm

**DOI:** 10.1002/ccr3.72978

**Published:** 2026-06-25

**Authors:** J. Curran Henson, Jordan H. Myers, Macy Cummins, Keyur Vyas, Michael Lowry

**Affiliations:** ^1^ Department of Internal Medicine University of Arkansas for Medical Sciences Little Rock Arkansas USA; ^2^ College of Medicine University of Arkansas for Medical Sciences Little Rock Arkansas USA; ^3^ Department of Pathology University of Arkansas for Medical Sciences Little Rock Arkansas USA; ^4^ Division of Infectious Disease University of Arkansas for Medical Sciences Little Rock Arkansas USA

**Keywords:** embolic stroke, granulomatous Aortitis, large‐vessel Vasculitis, Takayasu arteritis, thoracic aortic aneurysm

## Abstract

We describe the case of a 44‐year‐old female with a history significant for only hypertension who presented to the emergency department with symptoms of aphasia and difficulty concentrating, persisting for six hours. Work‐up revealed evidence of a multifocal infarct suggestive of embolic etiology, along with evidence of a large 6.0 × 6.0 ascending thoracic artery and simultaneous aneurysm of the transverse arch. The patient subsequently underwent open surgical repair with placement of a Dacron graft, which she tolerated well without complications. Surgical pathology was positive for granulomatous inflammation suggestive of autoimmune or infectious etiology, and the patient was readmitted post‐surgical discharge for an extensive work‐up, which was found to be negative. A new diagnosis of Takayasu arteritis (TA) was made. During her subsequent hospitalization, she developed an episode of unstable atrial fibrillation with rapid ventricular response and acute hypotension requiring medical ICU observation. She was started on high‐dose steroids, trimethoprim‐sulfamethoxazole prophylaxis, and amiodarone with a positive clinical response. She underwent a cardiac MRI to evaluate for evidence of myocardial infiltration, which was determined to be normal. The development of atrial fibrillation was considered a postoperative complication, and amiodarone was discontinued at eight weeks. The patient received a gradual steroid taper with initiation of daily low‐dose prednisone and methotrexate and made a full recovery.

## Graphical abstract

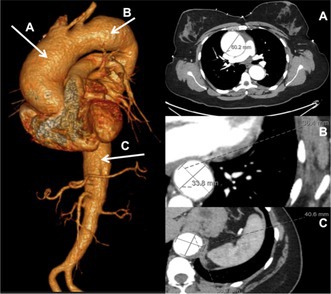

**Key Clinical Message**

This case emphasizes the importance of considering Takayasu Arteritis in patients with unexplained vascular abnormalities in atypical age groups. It highlights the need for comprehensive diagnostic work‐up, advanced imaging, and histopathology, alongside multidisciplinary management, including tailored medical and surgical interventions, to address severe complications like aneurysms, embolic stroke, and arrhythmias.

## 1 Introduction

Takayasu arteritis (TA) is an idiopathic large vessel vasculitis that typically involves the great vessels, including the aorta and its branches [1]. The underlying predisposition for the development of TA is not explicitly clear, but an etiology of autoimmune basis driven by both genetic and environmental factors is strongly suggested [2]. TA is known to present in occurrence with other comorbid necrotizing and non‐necrotizing autoimmune and rheumatological conditions, and even some immunodeficiency syndromes [2, 3].

The incidence of TA is known to be rare, affecting approximately 1–2 million people worldwide, with a predisposition to Asian women in their 20s. However, the true incidence of TA may be underestimated due to its rarity, frequent misattribution of symptoms to more common conditions, and delays in diagnosis [4, 5, 6]. These factors are associated with increased morbidity and mortality, often resulting from progressive vascular disease presenting as stenosis or aneurysm at the time of diagnosis [7].

Known as ‘pulseless’ disease, presentation of this pathology can occur with a wide range of symptoms, dependent upon the anatomic vascular bed involvement. These include claudication and diminished pulses from primary vascular involvement to hypertension (renal artery involvement), chest pain, abdominal pain, and neurologic dysfunction such as headaches, syncope, and dizziness secondary to intracranial vascular involvement. Physiological sequelae of this disease process are also varied, ranging from poor hemodynamics to large artery inflammation, intraluminal aneurysmal dilatation and compression, ischemic and embolic strokes, and severe progressive development of thoracic artery aneurysms. The concomitant occurrence of multiple secondary sequelae arising from this disease entity is not well documented and is presumed to be highly atypical. With these presuppositions in mind, we report a highly unusual case of TA in which the patient experienced the development of three aortic aneurysms (two thoracic, one abdominal) and a right frontal operculum embolic stroke prior to diagnosis. The age of our patient, at 44 years old, is also unique in the sense that most cases of TA are known to present in the second or third decade of life [2].

## 2 Case History and Examination

### 2.1 Clinical History

We describe the case of a 44‐year‐old female with a history significant for hypertension (not previously on medication) who presented to the emergency department with acute‐onset symptoms of aphasia and difficulty concentrating, persisting for six hours prior to presentation. The patient reported that she was preparing for work when she developed dizziness and nausea, followed by progressive difficulty with speech, describing “difficulty getting her words out.” She had never experienced similar symptoms previously, though she did report a history of intermittent headaches and blurry vision in the setting of elevated blood pressure readings, which she had been monitoring at home and noted to be consistently above 160/100 mmHg. She denied chest pain, dyspnea, syncope, focal motor weakness, or visual disturbances at the time of initial symptom onset, though she later reported mild chest discomfort and palpitations upon arrival to the emergency department. There was no prior history of autoimmune disease, vasculitis, or thromboembolic events. She endorsed a recent mild cough associated with an upper respiratory illness but denied fevers, chills, vomiting, abdominal pain, or shortness of breath.

### 2.2 Physical Examination

Vital signs upon presentation were significant for a systolic blood pressure of 172 mmHg and sinus tachycardia to 107–120 beats per minute. Neurologic examination demonstrated expressive aphasia without focal motor or sensory deficits. On further bedside evaluation performed by the vascular neurology service, the patient was awake, alert, and oriented to person, place, time, and situation. Motor examination revealed full strength (5/5) in all extremities without pronator drift, with normal tone and bulk. Sensory examination was intact to light touch and pinprick throughout. Reflexes were symmetric and normal, and coordination testing, including finger‐to‐nose and heel‐to‐shin, was intact without ataxia. Gait assessment was deferred. The patient's language disturbance was characterized by mild word‐finding difficulty and speech hesitancy, without dysarthria or impairment in comprehension. Symptoms were noted to be improving during hospitalization, and a formal speech‐language pathology evaluation confirmed improving expressive language function without dysarthria, with swallowing function within normal limits, and no evidence of dysphagia.

Cardiovascular examination was notable for tachycardia with regular rhythm, without murmurs, rubs, or gallops appreciated. No pulse deficits or inter‐arm blood pressure discrepancies were documented. There was no evidence of peripheral edema or vascular bruits on examination. On arrival to the CVICU, the patient remained hypertensive to 180/110 mmHg following transient discontinuation of esmolol but was otherwise calm and denied chest pain, dyspnea, or ongoing neurologic symptoms. The remainder of the physical examination was unremarkable.

### 2.3 Initial Clinical Impression and Differential Diagnosis

Given the patient's acute neurologic symptoms, the initial clinical concern was for a cerebrovascular accident. In the context of severe hypertension and tachycardia, additional considerations included hypertensive emergency and cardioembolic stroke. Following identification of a widened mediastinum on chest imaging, the differential diagnosis was broadened to include acute aortic syndromes such as aortic dissection, as well as large‐vessel vasculitis, infectious aortitis, and connective tissue disorders.

### 2.4 Investigations and Initial Hospital Course

Initial laboratory evaluation was significant for a mild leukocytosis to 10.03 K/uL and a high‐sensitivity troponin of 256.1 ng/L (reference < 14.9 ng/L), which subsequently increased to 622.3 ng/L on serial measurement. The electrocardiogram demonstrated sinus tachycardia with left axis deviation. Given the patient's acute‐onset aphasia and concern for cerebrovascular accident, computed tomography of the head (CTH) was obtained as an initial evaluation to assess for intracranial hemorrhage or large‐territory ischemic stroke and was found to be without acute intracranial process or evidence of large‐territory ischemic or hemorrhagic stroke. Given persistent neurologic symptoms despite a negative CT scan (Figure **1**A) and continued clinical concern for acute ischemic stroke, she subsequently underwent brain magnetic resonance imaging/angiography (MRI/MRA) for further evaluation. MRI/MRA (Figure **1**B) demonstrated a focal acute to early subacute infarct in the right posterior frontal lobe involving the frontal operculum with a punctate area of hemorrhagic transformation, as well as an additional punctate acute infarct in the right middle frontal gyrus. MRA demonstrated no hemodynamically significant stenosis or large vessel occlusion. Neurology and cardiology services were actively involved in the evaluation and management of this patient.

**FIGURE 1:**
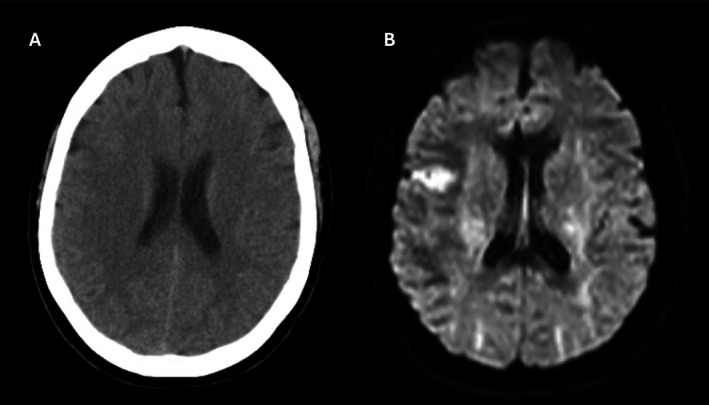
(A) Non‐contrast computed tomography (CT) of the head showing no evidence of acute intracranial hemorrhage or large‐territory infarction. (B) Diffusion‐weighted (DWI) magnetic resonance imaging (MRI) demonstrating a focal acute infarct in the right posterior frontal lobe involving the frontal operculum.

Continuous telemetry monitoring and electrocardiographic evaluation demonstrated a normal sinus rhythm without evidence of atrial fibrillation during the initial hospitalization. Transthoracic echocardiography revealed normal left ventricular systolic function (LVEF 55%–60%) with no regional wall motion abnormalities, no intracardiac thrombus, and no evidence of right‐to‐left shunt on color Doppler, although a bubble study was not performed. Mild aortic regurgitation was noted. As part of the stroke evaluation, carotid duplex ultrasonography demonstrated no hemodynamically significant stenosis bilaterally. Laboratory assessment revealed a low‐density lipoprotein (LDL) level of 72 mg/dL and glycated hemoglobin (HbA1c) of 5.4%.

A chest radiograph demonstrated a markedly widened mediastinum, prompting computed tomography angiography of the chest (Figure 2A–C), which revealed multifocal aneurysmal dilation involving the ascending aorta (Figure 2A), aortic arch (Figure 2B), and suprarenal abdominal aorta (Figure 2C) measuring 6.0 × 6.0 cm, 4.5 × 4.5 cm, and 4.1 × 3.7 cm, respectively, without evidence of dissection. Given these findings, she was admitted to the Cardiothoracic Surgery service for further surgical planning and was initiated on esmolol infusion in the emergency department, with nicardipine added in the CVICU for blood pressure control. She subsequently underwent left heart catheterization (LHC), which demonstrated no evidence of obstructive coronary artery disease. She then underwent open surgical repair of the thoracic aorta, consisting of resection and replacement of the ascending aorta, aortic arch, and proximal descending thoracic aorta utilizing a 30‐mm Dacron graft (elephant trunk technique). No intraluminal thrombus was identified on preoperative imaging or during intraoperative inspection. The remainder of her hospital course was uncomplicated, and she was discharged four days later in stable condition.

**FIGURE 2:**
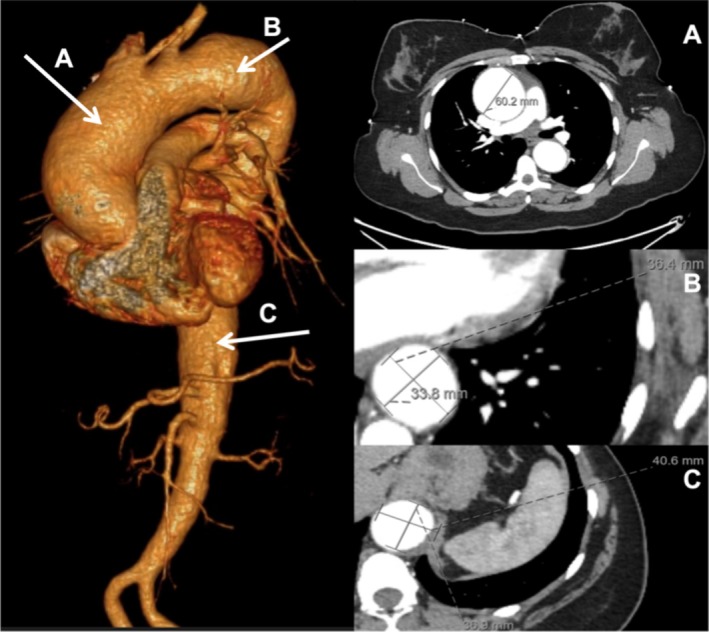
Multifocal aortic aneurysmal dilatation as evidenced by CT Angiography chest‐abdomen‐pelvis of the ascending aorta (A), aortic arch (B), and suprarenal abdominal aorta (C), measuring 6.0 × 6.0, 4.5 × 4.5, and 4.1 × 3.7 cm, respectively.

### 2.5 Readmission and Diagnostic Work‐Up

Report from the intraoperative surgical pathology was concerning for histopathological sections that revealed multiple granulomas predominantly within the muscularis layer. The central necrotic zones were predominantly surrounded by a large number of epithelioid histiocytes with occasional multinucleated giant cells (Figure 3B). The adventitia showed an intense multifocal lymphoplasmacytic infiltrate (Figure 3A). Myxoid changes of the vessel wall were also noted. When these histopathological findings were available, the patient was contacted to return to the emergency room at our institution for expedited admission and further work‐up.

**FIGURE 3:**
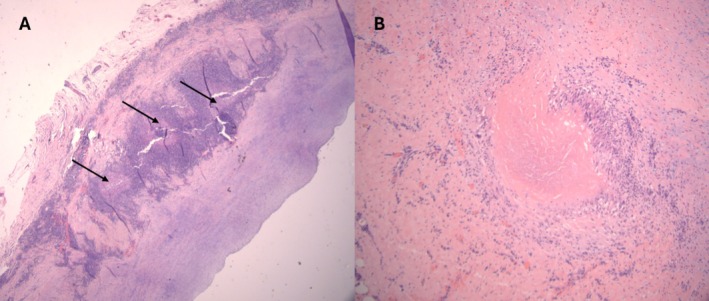
Histopathological sections obtained during thoracic aortic aneurysm resection and repair. (A) Arrows mark granulomas surrounded by intense lymphoplasmacytic infiltrate. (B) Granuloma with central necrosis.

At readmission, she was admitted to the infectious disease service, given concern from histopathologic findings of a likely autoimmune vs. infectious etiology of her granulomatous aortitis. Additional evaluation included HIV and syphilis serologic testing, which were not suggestive of active infection. Connective tissue disorders such as Marfan syndrome and Ehlers–Danlos syndrome were also considered; however, the absence of phenotypic features or family history, along with granulomatous histopathology, made these etiologies unlikely. She underwent a comprehensive diagnostic evaluation, as summarized in Table 1, with further diagnosis exclusion criteria specified within Table S1. Her ESR and CRP were elevated to 54 and 101.40, respectively. Autoimmune work‐up consisting of IgG4 titer, C3/C4 complement, ANA antibody screen, peripheral smear, HLA‐B27, and ANCA panel was all found to be negative. Concurrently, an infectious work‐up was performed, consisting of fungal elements (Coccidioides IgM/IgG, Histoplasma, and Blastomyces urine antigens), Brucella IgG, Bartonella IgG, tuberculosis testing, Coxiella IgM/IgG, HIV, and Tularemia IgM/IgG. Infectious work‐up was negative, albeit with a positive Tularemia IgG indicative of prior exposure. The histopathological sections were further evaluated with Ziehl–Nielsen staining, an immune‐stain panel, and Warthin stain, all of which were negative.

**TABLE 1:** Comprehensive differential diagnosis and exclusion criteria for Takayasu arteritis in the presented case, highlighting the systematic evaluation of autoimmune and infectious diagnostic considerations through clinical acumen, serological testing, and histopathological analysis to confirm the diagnosis and guide targeted management.

Pathology	Diagnosis Exclusion Criteria
Autoimmune/Rheumatologic	
Giant cell arteritis	Ruled out by age and histopathology findings
IgG4‐related disease	Excluded via negative IgG4 serology
Granulomatosis with polyangiitis (GPA)	Excluded by a negative ANCA panel
Sarcoidosis	Excluded by lack of systemic granulomas
Behçet's disease	No mucocutaneous lesions present
Systemic lupus erythematosus (SLE)	Negative ANA antibody screen
Rheumatoid arthritis (RA)	Negative rheumatoid factor (RF) and peripheral smear
Infectious	
Tuberculosis	Ruled out via stains and serology testing
Syphilis	Negative serologic testing (non‐reactive *Treponema Pallidum* Ab)
Fungal infections	Coccidioides, Histoplasma, and Blastomyces were excluded via antigen testing
Bartonella	Negative IgG serology
Brucellosis	Negative serology
Q fever ( *Coxiella burnetii* )	Negative IgM/IgG titers
Tularemia	Positive IgG deemed incidental/past exposure
Key Findings Supporting Exclusion	
Histopathology	Granulomatous aortitis without caseation or organisms; negative Ziehl‐Nielsen and Warthin‐Starry stains
Autoimmune markers	Negative HLA‐B27, C3/C4, ANA, ANCA
Infectious work‐up	No active infection identified despite positive Tularemia IgG (indicative of prior exposure)

### 2.6 Hospital Course and Final Diagnosis

During this hospitalization, she developed an episode of chest pain with unstable atrial fibrillation with rapid ventricular response and acute hypotension, with initial concern for postoperative endograft rupture. A STAT CTA was obtained that showed no evidence of rupture and confirmed patency of the stent. She underwent amiodarone loading with rapid conversion to normal sinus rhythm and was transferred to the medical ICU for observation. Her ICU course was uncomplicated, and she remained hemodynamically stable without further episodes of atrial fibrillation. She was transferred back to the general floor the following day.

Rheumatology was consulted and, given the extent of prior diagnostic evaluation, recommended no further work‐up. At their expert assessment, a diagnosis of Takayasu arteritis (TA) was made. Although the diagnosis in this case was established through histopathologic findings and exclusion of alternative etiologies, application of the 2022 American College of Rheumatology/European League Against Rheumatism (ACR/EULAR) classification criteria was further utilized to help further contextualize the diagnosis. The patient met entry criteria, including age < 60 years and imaging‐confirmed large‐vessel involvement. Additional criteria fulfilled included female sex (+1), imaging evidence of large‐vessel vasculitis (+2), and multi‐territory arterial involvement (+1), yielding a cumulative score of 4, just below the classification threshold of ≥ 5. The presence of granulomatous aortitis on histopathology, in conjunction with extensive exclusion of infectious and autoimmune etiologies, provides strong confirmatory evidence supporting the diagnosis beyond classification criteria alone.

Following the establishment of the diagnosis, management was directed toward immunosuppression and stabilization of her underlying inflammatory disease. She was started on high‐dose steroids (60 mg prednisone daily for three weeks), trimethoprim‐sulfamethoxazole prophylaxis, and continued on amiodarone with a positive clinical response. She was deemed stable for discharge five days post‐admission.

### 2.7 Outcome and Follow‐Up

She was followed closely in the clinic by both Cardiology and Rheumatology. She underwent a cardiac MRI to evaluate for evidence of myocardial infiltration, which was determined to be normal. The development of atrial fibrillation was considered a postoperative complication. Amiodarone was discontinued at eight weeks after Holter Monitoring revealed no further evidence of arrhythmogenic burden. The patient received a gradual steroid taper with initiation of daily low‐dose prednisone and methotrexate and made a full recovery.

## 3 Discussion

This case of TA presents a unique and complex clinical picture highlighting several important aspects of the disease's presentation, diagnosis, and management. The patient's age of onset is atypical as TA primarily has been shown to affect females in their 3rd and 4th decades of life, with the mean age of 30 [8]. This case therefore underscores the importance of considering TA even in older patients with suggestive symptoms, as cases have been reported in patients up to 75 years of age [8], emphasizing the need for vigilance across a broader age range. The concurrence of multiple aortic aneurysms and an embolic stroke is exceptionally rare in TA. While arterial stenosis is more common in TA, aneurysm formation occurs in approximately 25%–30% of cases [9, 10]. However, the presence of multiple aneurysms, as seen in this patient, is less frequent and may be suggestive of a more advanced disease course.

A comprehensive review by Keser et al. reported that aneurysms in TA most commonly affect the aorta and its major branches, with the potential for life‐threatening complications if left untreated [9, 11]. Cerebrovascular events, including strokes, occur in about 10%–20% of TA patients. Specifically, a study of 320 TA patients found that 20% had experienced either a stroke (65% of events) or a transient ischemic attack (35% of events), with 87% localizing to the carotid territory, often in association with multiple stenotic lesions and delayed diagnosis exceeding one year [12]. Stroke in TA patients is often ischemic in nature and associated with reduced cerebral blood flow to the brain secondary to stenosis of the aorta and its major branches [13, 14], but can also be embolic in nature, as seen in this case, where multifocal infarcts involving the right frontal operculum and an additional punctate lesion in the right middle frontal gyrus suggest a possible embolic mechanism.

From a mechanistic standpoint, and when interpreted within the TOAST classification framework, [15] this event is best categorized as a stroke of undetermined etiology. Large‐artery atherosclerosis was not supported by normal carotid duplex findings and the absence of flow‐limiting intracranial disease on MRA. A definite cardioembolic source was also not identified, given normal sinus rhythm on initial electrocardiographic and telemetry monitoring, absence of intracardiac thrombus on transthoracic echocardiography, and no evidence of right‐to‐left shunt on color Doppler. Small‐vessel disease was considered unlikely given the cortical distribution of infarction. Although the underlying large‐vessel inflammatory disease raises the possibility of vascular‐related stroke, no focal occlusive lesion or intraluminal thrombus was identified to establish a definitive alternative mechanism. Taken together, these findings suggest that while an embolic mechanism remains plausible based on the infarct pattern, the event is most accurately classified as cryptogenic.

Recent literature demonstrates that cerebrovascular complications of Takayasu arteritis are increasingly recognized across diverse global populations. In one report by Owino et al., a previously healthy young male without predisposing risk factors presented with a large‐territory ischemic stroke as the initial manifestation of Takayasu arteritis [16]. Similarly, Lamessa et al. reported a patient who also presented with ischemic stroke as the initial manifestation of previously undiagnosed Takayasu arteritis. This atypical finding emphasizes that delayed recognition is common due to the nonspecific and heterogeneous clinical presentation, often leading to diagnostic uncertainty early in the disease course [17]. Furthermore, Olalusi et al. described a case of a 33‐year‐old woman presenting with acute ischemic stroke accompanied by anterior ischemic optic neuropathy, hemiparesis, and expressive aphasia, highlighting a similar pattern of neurologic presentation to that seen in our case [18]. These results collectively highlight the need for monitoring of potential cerebrovascular events in TA patients and further underwrite the multifaceted complications that can arise from this unusual disease.

While not typically associated with TA, the development of atrial fibrillation in this patient is noteworthy. It may have been a postoperative complication or related to the extensive vascular inflammation. It has been reported that cardiac involvement in TA can occur with a prevalence as high as 50% [19], including the co‐occurrence of arrhythmias in up to 10% of cases [20], although the exact prevalence of atrial fibrillation is not well‐established. The absence of myocardial involvement as evidenced by cardiac MRI in our patient is reassuring that she will hopefully be at less risk of developing myocardial sequelae in the future as complications of her disease process.

Current management of TA is guided by expert‐defined guidelines, which emphasize the early initiation of high‐dose corticosteroids to induce remission, with adjunctive immunosuppressive agents being commonly employed as steroid‐sparing or permissive steroid‐tapering treatment strategies [21]. Commonly used regimens such as methotrexate, azathioprine, or mycophenolate mofetil are commonly utilized, and in patients with refractory or relapsing disease, tumor necrosis factor (TNF) inhibitors and interleukin‐6 (IL‐6) have demonstrated efficacy [21, 22]. While surgical intervention for aneurysm repair was crucial in this case for preventing potentially catastrophic bleeding complications, surgical or endovascular interventions for aneurysm repair and/or critical stenosis are ideally performed during periods of disease remission to reduce procedural risk [21, 22, 23]. The medical management discussed in this case reflects the current best practices in TA treatment; ergo, high‐dose induction corticosteroids followed by a gradual steroid taper and concurrent methotrexate.

## 4 Limitations

This case has several limitations that should be considered. While the multifocal pattern of infarction involving the right frontal operculum and right middle frontal gyrus suggests a possible embolic mechanism, a clear embolic source was not identified, and alternative explanations, such as hypoperfusion related to large‐vessel disease, cannot be completely excluded. Additionally, some clinical features included in Takayasu arteritis classification criteria, such as pulse deficits or inter‐arm blood pressure differences, were not documented, which limits the full application of these criteria. The classification criteria were applied retrospectively and interpreted alongside the broader clinical and histopathologic findings. Finally, as a single case report, these findings may not be broadly generalizable. Despite these limitations, the diagnosis is well supported by histopathologic evidence of granulomatous aortitis together with a thorough exclusion of infectious and autoimmune causes.

## 5 Conclusion

This case highlights how TA can present with multiple severe complications while not fully meeting established classification criteria, underscoring the importance of histopathologic confirmation in making the diagnosis. It also underscores the importance of maintaining a high index of suspicion for TA in patients with unexplained vascular abnormalities, the value of comprehensive diagnostic work‐up, including advanced imaging, and the need for a multidisciplinary approach to management. Particularly regarding the factors influencing the development of aneurysms vs. stenoses, this case adds crucial knowledge to the optimal approach in managing patients with multiple vascular complications. This case also highlights gaps in our understanding of TA, which can be partly ameliorated by the continued reporting of individual cases, thus contributing to the collective knowledge base and improving the diagnosis, management, and outcomes of this rare disorder.

## Author Contributions

**J. Curran Henson:** conceptualization, data curation, formal analysis, investigation, methodology, project administration, resources, supervision, validation, writing – original draft, writing – review and editing. **Jordan H. Myers:** data curation, investigation, methodology, project administration, writing – original draft. **Macy Cummins:** data curation, investigation, resources. **Keyur Vyas:** project administration, supervision, writing – review and editing. **Michael Lowry:** project administration, supervision, writing – review and editing.

## Funding

The authors have nothing to report.

## Consent

Written informed consent was obtained for this case report which ensured that the patient understood the purpose of the case report, the implications of participating, and the measures taken to protect their privacy and confidentiality. The patient provided voluntary consent for the publication of their case details.

## Supporting information


**Table S1:** Comprehensive laboratory, cardiovascular, and neuroimaging evaluation performed as part of stroke work‐up and etiologic assessment. Summary of general laboratory studies, vascular imaging, cardiac evaluation, and neuroimaging findings demonstrating absence of alternative stroke etiologies, including significant carotid disease, intracardiac thrombus, or persistent arrhythmia, with findings supporting an acute ischemic infarct without large vessel occlusion.

## Data Availability

Data sharing not applicable – no new data generated, or the article describes entirely theoretical research Data sharing not applicable to this article as no datasets were generated or analysed during the current study. All data provided within manuscript text and Supporting Information.
