# Supplementary material for: Atypical Case of Takayasu Aortitis Presenting as Embolic Stroke With Concomitant Massive Thoracic Artery Aneurysm
Source: Clin Case Rep. 2026 Jun 25;14(7):e72978. doi: 10.1002/ccr3.72978 (PMC13304228; doi:10.1002/ccr3.72978)
Supplement: Supplementary file 1 — Table S1: Comprehensive laboratory, cardiovascular, and neuroimaging evaluation performed as part of stroke work‐up and etiologic assessment. Summary of general laboratory studies, vascular imaging, cardiac evaluation, and neuroimaging findings demonstrating absence of alternative stroke etiologies, including significant carotid disease, intracardiac thrombus, or persistent arrhythmia, with findings supporting an acute ischemic infarct without large vessel occlusion. [file CCR3-14-e72978-s001.docx]

| GENERAL LABORATORY AND STROKE EVALUATION | |
| --- | --- |
| COMPLETE BLOOD COUNT (CBC) | Within normal limits; no evidence of anemia or leukocytosis beyond initial mild elevation |
| COMPREHENSIVE METABOLIC PANEL (CMP) | No clinically significant renal or metabolic abnormalities |
| LIPID PROFILE | No significant dyslipidemia identified |
| GLYCATED HEMOGLOBIN (HbA1c) | Within normal range; no evidence of diabetes mellitus |
| COAGULATION STUDIES (PT/INR, PTT) | Within normal limits; no evidence of coagulopathy |
| CAROTID DUPLEX ULTRASONOGRAPHY | No hemodynamically significant carotid stenosis |
| ECHOCARDIOGRAPHY | Normal left ventricular systolic function; no intracardiac thrombus or shunt identified |
| ELECTROCARDIOGRAPHY / TELEMETRY | Sinus tachycardia on initial presentation; no evidence of atrial fibrillation prior to stroke diagnosis. Transient postoperative atrial fibrillation with rapid ventricular response occurred during subsequent hospitalization |
| NEUROIMAGING (CT HEAD) | No acute intracranial hemorrhage or large-territory infarction |
| NEUROIMAGING (MRI/MRA) | Focal acute to early subacute infarct in the right posterior frontal lobe; no large vessel occlusion |

**Supplementary Table 1: Comprehensive laboratory, cardiovascular, and neuroimaging evaluation performed as part of stroke work-up and etiologic assessment.** Summary of general laboratory studies, vascular imaging, cardiac evaluation, and neuroimaging findings demonstrating absence of alternative stroke etiologies, including significant carotid disease, intracardiac thrombus, or persistent arrhythmia, with findings supporting an acute ischemic infarct without large vessel occlusion.
